# The impact of work pressure on decision-making effectiveness among department heads in faculties of educational sciences

**DOI:** 10.1371/journal.pone.0304584

**Published:** 2024-08-01

**Authors:** Zohair Al-Zoubi, Ahmed AlKaabi, Ahmad Qablan, Omar Bataineh, Hytham Bany Issa

**Affiliations:** 1 Department of Educational Foundations and Administration, College of Educational Sciences, The Hashemite University, Zarqa, Jordan; 2 Department of Foundations of Education, College of Education, United Arab Emirates University, Al Ain, United Arab Emirates; 3 Department of Curriculum and Instruction, College of Educational Sciences, The Hashemite University, Zarqa, Jordan; 4 Department of Curriculum and Instruction, College of Education, United Arab Emirates University, Al Ain, United Arab Emirates; COMSATS University Islamabad - Wah Campus, PAKISTAN

## Abstract

This study explores the dynamics of work pressure and decision-making effectiveness among university department heads within the context of Middle Eastern higher education. It aims to address a significant gap in the literature by answering five key questions: (1) What level of work pressure do department heads face as perceived by faculty members? (2) Does work pressure vary based on gender or years of experience? (3) How effective are department heads in decision-making? (4) Are there differences in decision-making effectiveness related to gender, university affiliation, or experience? (5) Is there a correlation between work pressure and decision-making effectiveness? A quantitative correlational research design was employed, gathering data from a questionnaire distributed to 205 faculty members. Correlational descriptive analysis revealed that department heads are subjected to high levels of work pressure but maintain a high level of decision-making effectiveness. However, significant differences in decision-making effectiveness were noted, with female faculty members performing better regardless of university affiliation or experience. A pronounced correlation was found between the intensity of work pressure and decision-making efficacy. The study underscores that department heads are under considerable work pressure, which could affect their productivity and, crucially, their decision-making processes. Universities are encouraged to take proactive steps to mitigate these pressures and promote a supportive, low-stress environment. Future research could extend this work by employing qualitative methods, including interviews and observations.

## Introduction

The increasing sensitivity of research and scholarship to political-economic contexts in higher education, both nationally and globally [1] signals evolving behaviors and values within academia. The competitive environment within universities intensifies pressure on faculty members. This pressure is particularly acute during economic downturns when downsizing can increase the workload for remaining staff. Active faculty engagement in various aspects of work is crucial for a university’s survival but often leads to increased work-related stress.

Work stress in industrial-organizational psychology is defined as a response to workplace stimuli that can lead to negative outcomes for those exposed. University faculty members, in particular, face extensive responsibilities, including research, teaching, and community service, all of which have become more demanding [2]. These responsibilities, coupled with family, social, and community obligations, can lead to high stress levels. This stress can result in reduced job satisfaction, increased anxiety, depression, turnover intentions, and various disorders that hinder adaptation to changing work conditions [3].

Work stressors vary across environments and can stem from organizational factors (e.g., workload, salary), functional factors (e.g., the physical work environment, decision-making, feedback), and personal factors (e.g., personality, psychological and physical health). While extensive research has explored the impact of stress on memory, less is known about its effects on decision-making abilities, especially under stress [4, 5]. This gap is more pronounced in the context of Middle Eastern higher education.

Several studies have examined work pressure, investigating workplace conditions [6], assessing work pressure in universities [7, 8], and exploring its impact on work performance [9]. However, the direct correlation between work pressure and decision-making in Middle Eastern academic settings, especially among heads of academic departments in Jordanian educational science faculties, remains under-researched. This study aims to fill this void.

This research investigates work pressure and decision-making effectiveness among university department heads. It centers on these primary inquiries: (1) What level of work pressure do department heads face as perceived by faculty members? (2) Does work pressure vary based on gender or years of experience? (3) How effective are department heads in decision-making? (4) Are there differences in decision-making effectiveness related to gender, university affiliation, or experience? (5) Is there a correlation between work pressure and decision-making effectiveness? This study considers work pressure and decision-making as dependent variables, with gender and experience as independent variables. Its significance lies in enhancing our understanding of the challenges facing university department heads and the impact on their decision-making efficiency. The results will provide insights for universities to develop leadership support structures.

By exploring potential disparities in work pressure and decision-making effectiveness across demographics, this research contributes to supporting diversity and equality in academic leadership. Understanding the decision-making capabilities of department heads is vital for institutional governance and academic program management. This study adds to the body of knowledge by focusing on the unique experiences and strategies of university department heads, thus addressing a gap in our understanding of their professional challenges. The following section presents a theoretical background that examines the interplay between stress and decision-making in higher education, incorporating empirical research from both global and national contexts.

## Literature review

Work pressure and decision-making capabilities are pivotal to the efficiency and success of higher educational institutions. These institutions operate within a dynamic environment that presents department heads and administrators with complex challenges and decisions. Effective management of work pressures and adept decision-making are essential as they significantly influence educational quality, staff welfare, and institutional success. This section delves into work pressure and decision-making within higher education, underscoring their role in shaping the academic landscape and promoting a conducive learning and working climate.

### Theoretical background

#### Work pressure

Workplace pressure is ubiquitous across professions, and it affects fields from physics to educational administration. It arises when job demands alter an individual’s psychological or physiological state, impeding normal functioning [10]. Academic administrators, particularly department heads, often face daunting tasks that affect their managerial efficiency [11]. Balancing multiple managerial and teaching responsibilities can exacerbate stress levels and lead to physical, psychological, and emotional strain [6]. It is worth noting that, regardless of the excessive workload, motivation and rewards also play a crucial role in individual behaviors [12–14].

In a qualitative study, [6] found that university faculty members identified workload, job conditions, job security, promotion delays, and unsupportive environments as primary stressors. The study suggested that fostering a supportive work environment and offering professional development opportunities could help alleviate stress. [8] noted the significant impact of faculty work pressures. [15] explored the physical and psychological risks associated with increasing academic demands, including competitive research, teaching responsibilities, fundraising efforts, and administrative tasks. Collectively, these studies highlight the complex and multifaceted nature of work pressures in academia and their far-reaching consequences.

A recent trend sees professors increasingly burdened with administrative responsibilities without corresponding benefits. [7] found moderate administrative work pressure among leaders at Jordanian public universities. [16] reported that 60% of UK teaching and research staff disliked administration, and 49% felt their workload adversely affected their personal life. Furthermore, 24% experienced intolerable stress levels. Excessive administrative duties, alongside teaching and research, detrimentally impact professors’ psychosocial well-being and coping mechanisms, which indicates workload as a stress factor potentially harmful to university organization [17]. However, a healthy environment with well-managed leadership can boost individual satisfaction [18].

In their study, [9] examined the relationship between work pressure and work quality in academic leadership at Jordanian public universities. Using a questionnaire targeting 28 fields and involving 80 academic leaders from four universities, they found a significant link between work pressure and educational quality. The study identified significant differences in work pressure levels among leaders, related to experience and job title.

Furthermore, [19] assessed the mental health of 949 teachers and found that many had substantial mental health issues related to stress, including concentration difficulties, sleep disturbances, and depressive symptoms. Addressing these issues, [20] argued that the success of higher education institutions hinges on the well-being of faculty members. He advocated for continuous development of faculty, management, and students to enhance performance and achieve institutional goals while underscoring the need to alleviate workplace pressures.

#### Decision-making

Decision-making varies significantly across professions. In higher education, this responsibility is particularly complex and demanding. Academic department heads at universities face some of the most stressful situations on campus. They are tasked with balancing responsibilities toward faculty, staff, and management, requiring reliance on their confidence, identity, and principles to make well-informed decisions [21]. Stress can profoundly impact decision-making. Under stress, cognitive impairments such as diminished attention, memory, and problem-solving abilities may arise, which can lead to impulsive decisions or decision avoidance [22].

Work-related stress can trigger emotional responses like fear, anger, and anxiety, which can influence decision-making. Stress may lead to decisions based on irrational or emotionally driven factors. Stress impacts decision-making at both routine and critical levels and extends to neural responses [22]. A study by [23] aimed to assess the efficacy of decisions made by the academic department boards in Jordanian universities. They developed a questionnaire comprising 52 items, which they distributed to faculty members across various Jordanian universities. The findings suggested that the faculty members perceived the decisions of these academic department boards as highly effective. This perception was particularly pronounced in public universities compared to government and private institutions.

[24] conducted research to evaluate decision-making effectiveness within the Faculties Boards and Departmental Councils of the Applied Colleges at Saudi Arabian Universities. The study involved 60 participants and revealed a moderate level of decision-making efficiency. Notably, the Department Heads were instrumental in supporting the quality assurance policies and procedures of the Allied Colleges. The authors recommended that the Faculties Boards and Departmental Councils should improve their decision-making processes to enhance overall effectiveness and efficiency.

[25] undertook a study to examine the impact of knowledge management on enhancing administrative decisions among academic department heads in Jordanian universities. Employing a descriptive survey methodology, they administered a questionnaire to 84 department heads. The results indicated that knowledge management significantly contributed to improving the administrative decisions of these heads, particularly in Jordan’s northern region. The study found no significant gender-based or university-based differences. However, differences were noted in relation to academic rank, favoring those in professorial positions; and years of experience, favoring individuals with over 10 years of experience.

### The Yerkes-Dodson law model

**Fig 1:**
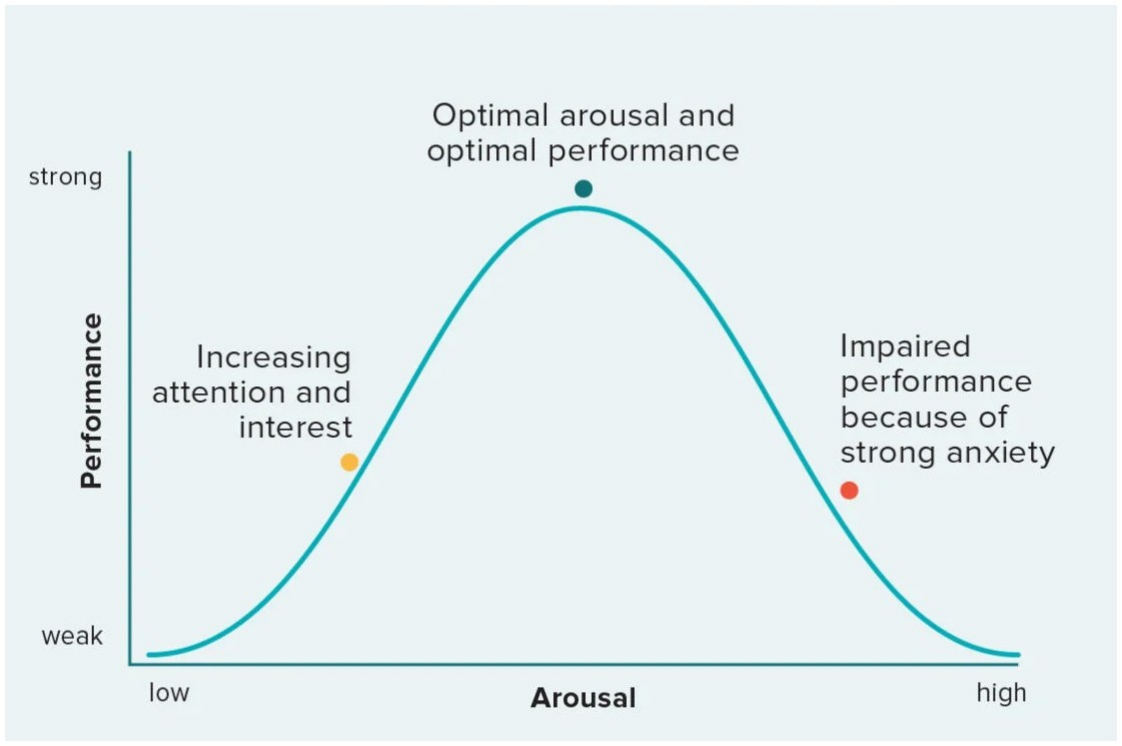
The Yerkes-Dodson law illustrates the relationship between stress and performance.

The Yerkes-Dodson law is a well-known psychological principle that describes the relationship between arousal (or stress) and performance. According to this model, there is a curvilinear relationship between the two, suggesting that performance increases with arousal or stress up to a certain point, after which it begins to decline [26]. At low levels of stress, individuals may lack the motivation or arousal needed to perform well, leading to suboptimal performance. As stress levels increase, so does performance, as individuals become more focused and energized. This is often referred to as the "optimal arousal" zone, where individuals are at their peak performance levels. However, beyond this optimal level, further increases in stress can lead to a decline in performance. This is because excessive stress can lead to feelings of being overwhelmed, which can impair cognitive function and decision-making abilities [26]. See Fig 1 below, which illustrates the principles of the Yerkes-Dodson law.

In essence, the Yerkes-Dodson law suggests that there is an optimal level of stress that leads to peak performance, and that both too little and too much stress can be detrimental to performance. In the workplace, the Yerkes-Dodson law can have important implications for understanding how stress affects decision-making. Employers may need to consider how to manage stress levels among employees to ensure that they are operating within the optimal arousal zone for peak performance. This could involve implementing stress-reduction strategies, providing resources for coping with stress, and creating a supportive work environment.

In higher education, faculty members often experience varying levels of stress, which can impact their decision-making and overall performance. According to the Yerkes-Dodson law, there is an optimal level of stress that can enhance performance, but beyond that point, performance may decline [26].

The literature extensively examines the detrimental impact of workplace stress on faculty members and academic administrators. It also addresses the complexities of decision-making within academic environments. However, despite separate examinations of work pressure and decision-making, there is a noticeable gap in the literature regarding their interrelation. While studies explore the adverse effects of work pressure on faculty members [6–8, 10, 11, 15] and the complexities of decision-making in academic settings [23, 25], there is limited exploration of how work pressure directly affects decision-making capabilities.

Therefore, a potential research gap exists in the literature regarding the correlation between work pressure and decision-making in higher education contexts. Investigating this correlation could yield insights into how stressors associated with work pressure influence the decision-making effectiveness of academic department heads and faculty members. Addressing this gap could enhance understanding of the complex relationship between work pressure and decision-making in higher education department heads.

## Methods

To achieve the objectives of the current study, the researchers employed a descriptive, correlative survey method. This approach is designed to elucidate the relationships between two or more variables without asserting causality. It involves the collection and analysis of data on at least two variables to ascertain if a linkage exists between them [27]. The suitability of this method for the present study lies in its capacity to effectively gather data on the phenomena under investigation from a substantially large number of participants. The descriptive component of the methodology enabled the researchers to capture a comprehensive picture of the existing scenario that encompassed the extent of work pressure experienced by department heads and the degree of effectiveness in decision-making. Conversely, the correlative element of the survey facilitated the examination of potential relationships between these variables. This examination was crucial in exploring whether a correlation existed between work pressures and decision-making effectiveness. Furthermore, this method allowed for the collection of robust data to form a solid foundation for the study’s conclusions and recommendations.

### Population and sample

The study’s population encompasses all faculty members within the faculties of educational sciences at Jordanian universities. The 2021 Statistics from the Ministry of Higher Education and Scientific Research revealed that this group comprised 1,435 faculty members. The study’s sample is drawn from this population, consisting of 205 faculty members, which represents 15% of the total population. The distribution of the study’s sample is presented in Table 1 below.

**Table 1:** Variable-Based distribution of study sample.

Variable	Category	Number of Faculty	Percentage
**Gender**	Female	68	% 33.2
	Male	137	% 66.8
**University**	State	139	% 67.8
	Private	66	% 32.2
**Years of Experience**	Less than 5 Years	31	% 15.1
	From 5–10 Years	33	% 16.1
	More than 10 Years	141	% 68.8
**Total**		205	% 100

### Instruments for data collection

Two questionnaires were specifically designed for the present study. The first questionnaire delves into the intricate work pressures encountered by department heads within educational sciences faculties [7]. The second questionnaire was meticulously crafted to gauge the effectiveness of decision-making among these department heads. Both questionnaires adopted a five-point Likert scale, wherein respondents indicated their level of agreement or disagreement using the following scale: 5 = Strongly Agree, 4 = Agree, 3 = Neutral, 2 = Disagree, and 1 = Strongly Disagree. To evaluate the scores, a rating plan based on the average was employed, which categorized the results into three groups: low (1–2.33), medium (2.34–3.67), and high (3.68–5).

To ensure the validity of the questionnaires, an expert committee examined the relevance, clarity, and linguistic accuracy of the questionnaires’ items in their initial form. Panel members were given the opportunity to suggest any necessary additions, deletions, or replacements for the questionnaire items. After considering valuable feedback from the experts, the questionnaire was thoroughly reviewed, and any needed adjustments were made to the final form. Cronbach’s alpha coefficient was utilized to gauge the internal consistency and reliability of the elements within the two scales. The elements pertaining to work pressure yielded a coefficient value of 0.919, while the elements aimed at effective decision-making achieved a coefficient value of 0.897 (see Table 2).

**Table 2:** Faculty pressures and decision-making: Cronbach’s alpha constancy factor.

Area	Number	Cronbach’s Alpha Constancy Factor
**Community Pressures**	8	0.827
**Student Pressures**	7	0.819
**Pressures on Teaching Staff**	8	0.716
**Occupational Pressures**	8	0.874
**Overall Work Pressures**	31	0.919
**Effectiveness of Decision-Making**	18	0.897

### Data collection

Data for the study were collected using electronic forms, which provided a convenient and efficient means of data collection. Faculty members who met the study’s criteria were invited to participate, and their willingness to take part was confirmed through a consent form. After obtaining consent, participants were then directed to respond to a series of questions that encompassed both their demographic information and the elements of the questionnaire. This approach allowed for systematic data gathering while maintaining participant confidentiality and convenience. Data collection occurred from October 2022 to December 2022.

### Data analysis

The 28th version of SPSS software was used for data analysis. Descriptive statistics, including mean and standard deviation (SD), summarized the data. Inferential statistics, such as correlation analysis and three-way ANOVA, explored relationships and patterns. The Pearson correlation coefficient assessed the relationships’ strength and direction, and the three-way ANOVA tested the interaction effect of gender, university, and years of experience on the dependent variable (degree of work pressures). This comprehensive analytical approach provided valuable insights for examining the research questions.

## Results

To address the first research question concerning the degree of work pressures experienced by academic department heads, the average and standard deviation of the overall responses were calculated. The findings revealed an average rating of 3.67 out of 5.00, accompanied by a standard deviation of 0.87. Based on the established criteria, these results suggest a high level of work pressure. In terms of specific aspects of work pressures, items related to the local community exhibited the highest average rating (3.81, SD = 0.91), indicating considerable pressure. Work pressures associated with students yielded an average rating of 3.73 (SD = 0.88), also suggesting a high level of pressure. For faculty members’ work pressures, the average rating was 3.60 (SD = 0.92), reflecting an intermediate level of pressure. Lastly, occupational stress areas received an average rating of 3.54 (SD = 0.94), which denoted a moderate level of work pressure.

To address the second research question concerning potential statistically significant differences in work stress among faculty members within faculties of educational sciences at Jordanian universities, an analysis of variance (ANOVA) was conducted based on gender, university type, and years of experience. The results indicated no statistically significant differences at the α = 0.05 significance level in the degree of work stress faced by heads of academic departments in educational science colleges, according to the perceptions of the study participants. This conclusion is supported by the calculated F-values of 0.958 for gender, 0.192 for university type, and 0.184 for years of experience, with corresponding p-values of 0.329, 0.662, and 0.832, respectively, as detailed in Table 3 below.

**Table 3:** Analysis of variance (three-way ANOVA) results: Degree of work pressures faced by academic department heads in educational science colleges.

Source of Variation	Total of Squares	Average of Squares	F-Value	Significance Level
**Gender**	0.727	0.73	0.958	0.329
**University**	0.146	0.15	0.192	0.662
**Years of Experience**	0.279	0.14	0.184	0.832
**Error**	151.839	0.76		
**Total**	152.882			

In response to the third question regarding the effectiveness of decision-making by the heads of academic departments in the faculties of educational sciences, the averages and standard deviations were calculated. The results showed an average of 3.89 with a standard deviation of 0.70, which indicated a high average level of effectiveness.

Regarding the fourth research question, which examined the influence of gender, university, and years of experience on the effectiveness of decision-making, an analysis of variance was conducted. This analysis assessed the statistical significance of these variables. The results showed significant differences at a significance level of α = 0.05 in the decision-making effectiveness of the heads of academic departments based on gender. Specifically, the calculated F-value was 4.47 with a significance level of 0.04, favoring female teaching staff members. This suggests that female staff exhibited higher decision-making effectiveness compared to their male counterparts. Conversely, no statistically significant differences were observed in decision-making effectiveness based on university and years of experience. The F-values for university and years of experience were 2.50 and 0.57, respectively, with corresponding significance levels of 0.12 and 0.57. These findings indicate that university affiliation and years of experience did not significantly impact the decision-making effectiveness of the heads of academic departments. The summarized results are presented in Table 4.

**Table 4:** Analysis of variance (three-way ANOVA) results: Level of decision-making effectiveness among department heads in educational science colleges.

**Source of Variation**	**Total of Squares**	**Average of Squares**	**F-Value**	**Significance Level**
**Gender**	2.14	2.14	4.47	0.04*
**University**	1.20	1.20	2.50	0.12
**Years of Experience**	0.55	0.27	0.57	0.56
**Error**	95.81	0.48		
**Total**	3208.10			

*Note*. Asterisk (*) denotes a p-value that is less than 0.05.

The fifth research question investigated the correlation between work pressure and decision-making effectiveness among heads of academic departments in the educational sciences faculties of Jordanian universities. To address this, Pearson’s correlation coefficient was employed. The results demonstrated a significant positive correlation (r = 0.680, p < 0.01) between work pressure and decision-making effectiveness as perceived by the faculty. Additionally, the correlation coefficients between specific areas of work stress (community work pressures, student work pressures, faculty work pressures, occupational work pressures) and decision-making effectiveness were also significant (r = 0.620, 0.627, 0.665, 0.669, respectively, all p < 0.01). These findings indicate a strong correlation between these variables, as detailed in Table 5.

**Table 5:** Pearson correlation: Work stress and decision-making effectiveness in Jordanian educational sciences faculties.

Variable	Statistical Scale	Effectiveness of Decision-making
**Community Pressures**	Pearson Coefficient	0.620
	Significance Level	0.00*
**Student Pressures**	Pearson Coefficient	0.627
	Significance Level	0.00*
**Pressures on Teaching Staff**	Pearson Coefficient	0.665
	Significance Level	0.00*
**Occupational Pressures**	Pearson Coefficient	0.669
	Significance Level	0.00*
**Overall Work Pressures**	Pearson Coefficient	0.680
	Significance Level	0.00*

*Note*. Asterisk (*) denotes a p-value that is less than 0.05.

## Discussion

In our study examining faculty perceptions of work pressures faced by department heads in educational sciences faculties at Jordanian universities, we observed high levels of stress, consistent with findings by [8, 16] but contrasting with [7], who reported moderate stress levels. This variation in stress can be attributed to the multifaceted and demanding roles of department heads, which include managing departmental affairs and decision-making, known to contribute to elevated stress levels.

When exploring whether work pressure levels vary significantly based on factors like gender and years of experience, our findings revealed no significant differences, which is in contrast with [9], who found high significance based on years of experience. This absence of significant variation in our study could be due to increased responsibilities and expectations, such as high student enrollment and the requirement for Scopus-indexed research publications, which impact department heads regardless of experience. The excessive administrative, teaching, and research obligations underline the importance of workload as a stress factor, potentially impacting the well-being and coping mechanisms of university professors.

Regarding the decision-making effectiveness among department heads, our results indicated a high level of effectiveness, aligning with [23] and contrasting with [24]. This high effectiveness in decision-making could be influenced by a cooperative environment within academic departments, where faculty members actively participate in various responsibilities, fostering a supportive atmosphere for effective decision-making.

As for the influence of gender, university affiliation, and years of experience on decision-making effectiveness, our study found statistically significant differences based on gender, with female department heads exhibiting higher effectiveness. This finding, contrasting with [25], suggests the importance of active engagement in decision-making. However, our study did not find significant differences based on university affiliation or years of experience, which indicates a level of uniformity in decision-making processes across departments.

Lastly, we examined the potential correlation between work pressure and decision-making effectiveness and found a strong relationship, suggesting that high levels of work pressure negatively impact decision-making effectiveness among department heads. This is indicative of the physical or psychological impacts that can disrupt a decision-maker’s balance and lead to increased tension and impaired decision-making abilities, as supported by studies such as [5, 19]. This finding also aligns with the Yerkes-Dodson law, which posits that there is an optimal level of arousal or stress for performance, and beyond that point, performance can decline [26]. In the context of decision-making, excessive work pressure may push individuals beyond this optimal level, leading to impaired decision-making abilities.

## Conclusion

This study has delved deeply into the dynamics of work pressures and their impact on the decision-making efficacy of academic department heads in Jordanian universities. A detailed analysis of a survey involving 205 faculty members has yielded significant insights. These insights not only confirm the intense work pressures these leaders endure but also illuminate the nuances of their decision-making processes. Our research uncovers a complex interplay between work pressures and decision-making effectiveness and highlights the resilience and adaptability of department heads under substantial professional strain. Additionally, the study reveals intriguing patterns in how gender and experience influence decision-making efficiency, and it offers a refined understanding of leadership within the challenging milieu of higher education. These discoveries pave the way for further exploration of practical implications and future research avenues, which may serve as a foundation for targeted interventions and policy modifications aimed at enhancing the well-being and effectiveness of academic leaders in this region.

### Implications for practice

The study’s findings shed light on the fact that department heads contend with elevated work pressure, which may potentially affect their overall productivity and, more notably, their decision-making processes. In recognizing the significant work pressures faced by department heads, universities should take proactive measures to alleviate these pressures and promote a healthy stress environment. This can be achieved by providing administrative support to department heads in managing various responsibilities, such as departmental boards, affairs, meetings, and correspondence. By lightening their workload and offering support, universities can help reduce stress levels and enable department heads to focus more effectively on decision-making. In addition to administrative support, fostering a supportive and collaborative culture within academic departments is crucial. Encouraging the active participation of faculty members in decision-making processes and sharing administrative tasks can create a sense of collective responsibility and alleviate the workload on department heads. This collaborative approach not only enhances decision-making effectiveness but also promotes a positive work environment where individuals can rely on each other for support and assistance.

Furthermore, by understanding how stress impacts decision-making according to the Yerkes-Dodson law, universities can tailor their support and development programs to better meet the needs of department heads, ultimately leading to a more efficient and effective academic environment. By enhancing their skills in decision-making, time management, and stress management, universities can empower them to navigate their roles more effectively. Equipping them with effective strategies and tools can significantly contribute to their decision-making capabilities, ultimately benefiting the entire department and enhancing overall performance. Effective communication channels within departments are also vital for reducing work pressures and facilitating decision-making processes. Implementing streamlined information flow, preventing information overload, and ensuring clear and efficient communication can help department heads make well-informed decisions in a timely manner. By providing the necessary infrastructure and support for effective communication, universities can empower them to handle their responsibilities more efficiently and mitigate unnecessary stress.

Promoting work-life balance is essential for department heads’ well-being and decision-making effectiveness. Universities can organize workshops or training sessions focused on stress management techniques, such as mindfulness, time management, relaxation techniques, and provide practical tips and strategies that faculty can incorporate into their daily routines. Encouraging regular breaks, vacations, and opportunities for relaxation and rejuvenation can prevent burnout and enhance overall job satisfaction. By prioritizing work-life balance, universities demonstrate their commitment to the well-being of department heads, fostering a healthier and more productive work environment. Lastly, creating a supportive network and community of practice among department heads can be highly beneficial. This platform allows for the exchange of experiences, challenges, and best practices among peers, reducing feelings of isolation and stress. By encouraging collaboration and knowledge-sharing, universities can tap into the collective wisdom of department heads and promote continuous improvement and enhancement in decision-making effectiveness across departments.

In implementing these recommendations, universities can create a healthier and more supportive environment for department heads, ultimately enhancing their decision-making effectiveness and overall well-being. By recognizing the importance of work-life balance, fostering collaboration, providing professional development opportunities, improving communication channels, and cultivating a supportive network, universities can empower department heads to thrive in their roles and contribute positively to the success of their departments.

### Future research

In considering future research, several promising avenues emerge. First, replicating this study using a qualitative methodology, which includes interviews and observations, could be enlightening. Such a method would allow for the gathering of more nuanced data regarding work pressures and decision-making processes, providing deeper insights into the lived experiences of university department heads. In addition, future studies could benefit from a broader approach, such as conducting comparative analyses among universities in neighboring countries. This comparative study would be crucial in identifying the factors that influence work pressures in different educational environments, thereby highlighting regional and contextual differences. Furthermore, an in-depth exploration of burnout among university department heads is essential. Investigating the potential relationship between work pressure and burnout, and its effects on decision-making efficiency, could offer vital insights into the well-being and effectiveness of academic leaders.

### Limitations

The sample size of 205 faculty members in this study, though substantial, constitutes only a fraction of the total faculty population of 1,435 in the educational sciences faculties at Jordanian universities. While this sample might be adequate for preliminary insights, its size could limit the broader applicability of the findings. To enhance the external validity, future studies could benefit from a larger and more diverse sample. Additionally, the study’s reliance on self-reported data from faculty members may introduce response bias. This bias could occur if participants provide responses they perceive as socially desirable or if they inaccurately assess their own work pressures and decision-making effectiveness. Such discrepancies might impact the accuracy and reliability of the study’s outcomes.

## Supporting information

S1 Data (SAV)
